# Supplementary material for: Diet Composition, Glucose Homeostasis, and Weight Regain in the YoYo Study
Source: Nutrients. 2021 Jun 30;13(7):2257. doi: 10.3390/nu13072257 (PMC8308328; doi:10.3390/nu13072257)
Supplement: Supplementary file 1 [file nutrients-13-02257-s001.zip › nutrients-1241181-supplementary.pdf]

Supplemental Table S1. Subject characteristics and anthropometrics, glucose homeostasis parameters and dietary intakes (mean (SD)) at the different time points of the study (baseline, end of WL, end of CWM and FU) in participants with FPG < 5.6 mmol/L and with FPG ≥ 5.6 mmol/L.

	**FPG (mmol/L)**	***n***	**Baseline**	**end of WL**	**end of CWM**	**FU**	***p* value***
**Age (year)**	< 5.6	44	50.3 (8.9)				
	≥5.6	13	54.4 (9.5)				
**Sex (male/female)**	< 5.6	44	19/25				
	≥5.6	13	8/5				
**Height (cm)**	< 5.6	44	171.3 (9.0)				
	≥5.6	13	175.5 (8.3)				
**Weight (kg)**	< 5.6	37	90.7 (9.6)	82.0 (8.8)^{a}	81.8 (9.1)^{a}	86.5 (9.5)^{a,c,d}	<0.001
	≥5.6	10	97.0 (9.3)	88.4 (8.7)^{a}	87.6 (8.9)^{a,c}	91.9 (11.3)^{b,d}	<0.001
**BMI (kg/m^{2})**	< 5.6	37	30.9 (2.4)	27.39(2.5)^{a}	27.9 (2.6)^{a}	29.5 (2.9)^{a,c,d}	<0.001
	≥5.6	10	31.6 (1.9)	28.8 (1.9)^{a}	28.5 (2.0)^{a,c}	29.9 (2.0)^{b,,d}	<0.001
**Body fat%**	< 5.6	37	39.8 (8.9)	34.5 (10.8)^{a}	33.7 (10.7)^{a,c}	37.0 (10.3)^{a,c,d}	<0.001
						34.4 (10.1)	
	≥5.6	10	38.1 (9.7)	33.5 (10.8)^{a}	32.2 (11.1)^{a,c}		<0.001
**FPG (mmol/L)**	< 5.6	37	5.09 (0.27)	4.84 (0.32)^{a}	4.95 (0.39)	4.86 (0.37)^{a}	<0.001
	≥5.6	10	5.91 (0.29)	5.35 (0.47)^{a}	5.48 (0.29)^{a}	5.42 (0.39)^{a}	0.002
**FPI (μIU/mL)**	< 5.6	37	15.1 (6.0)	11.1 (4.7)^{a}	13.0 (5.4)^{a,c}	12.7 (8.5)	0.002
	≥5.6	10	18.0 (6.1)	10.4 (2.4)^{a}	11.2 (3.6)^{b}	12.7 (8.4)	0.010
**HOMA-IR**	< 5.6	37	3.44 (1.45)	2.42 (1.14)^{a}	2.90 (1.44)^{b,c}	2.62 (1.74)	<0.001
	≥5.6	10	4.71 (1.60)	2.51 (0.67)^{a}	2.77 (0.90)^{a}	3.10 (2.29)	0.007
**Energy intake**	< 5.6	42	9373 (3305)	NR	7268 (1840)^{a}	7453 (2349)^{a}	0.001
**(kJ/day)**					8179 (2358)	7996 (2286)	
	≥5.6	8	9738 (3406)	NR			0.285
**Carbohydrate**	< 5.6	42	46.4 (8.1)	NR	48.5 (7.0)	48.7 (7.4)	0.056
**intake (energy%)**	≥5.6	8	44.3 (6.4)	NR	46.6 (5.8)	47.6 (8.5)	0.471
**Fat intake**	< 5.6	42	35.9 (7.2)	NR	30.1 (5.6)^{a}	32.4 (6.3)^{b}	0.000
**(energy%)**	≥5.6	8	32.5 (2.1)	NR	32.5 (2.1)	33.5 (3.5)	0.752
**Protein intake**	< 5.6	42	17.3 (4.0)	NR	21.1 (4.3)^{a}	18.9 (4.2)^{d}	0.000
**(energy%)**	≥5.6	8	18.0 (4.5)	NR	19.4 (2.4)	17.9 (3.3)	0.593
**Fibre intake**	< 5.6	42	10.4 (3.7)	NR	14.7 (4.2)^{a}	12.7 (3.5)^{a}	0.000
**(g/1000kcal)**	≥5.6	8	9.6 (4.7)	NR	10.8 (2.4)	12.8 (3.7)	0.190

WL = weight loss; CWM = controlled weight maintenance; FU = follow-up; *n* = number of subjects; BMI = body mass index; FPG = fasting plasma glucose; FPI = fasting plasma insulin concentration; HOMA-IR = homeostatic model assessment of insulin resistance; * *p* value of repeated measurements ANOVA; NR = not recorded. Post-hoc pairwise comparison (Bonferroni corrected): ^{a} $p < 0.01$ vs baseline; ^{b} $p < 0.05$ vs baseline; ^{c} $p < 0.05$ vs end of weight loss; ^{d} $p < 0.01$ vs end of controlled weight maintenance.

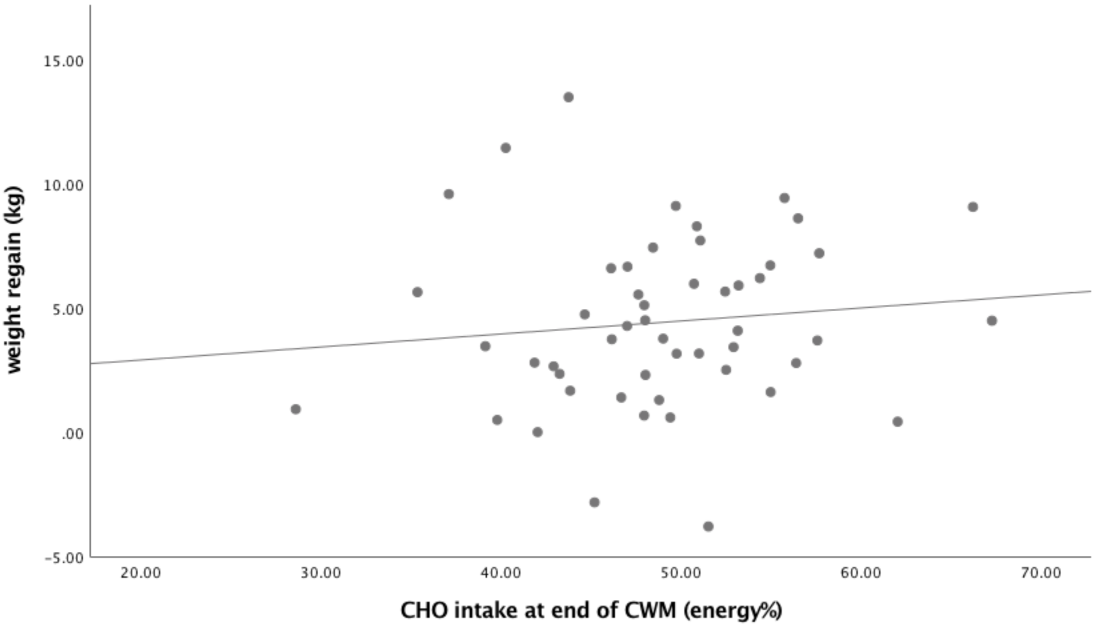

Supplemental Figure S1. Association between dietary carbohydrate (CHO) intake at the end of CWM and weight regain (n = 48). Regression analysis: B = 0.052, SE 0.068, p = 0.442.

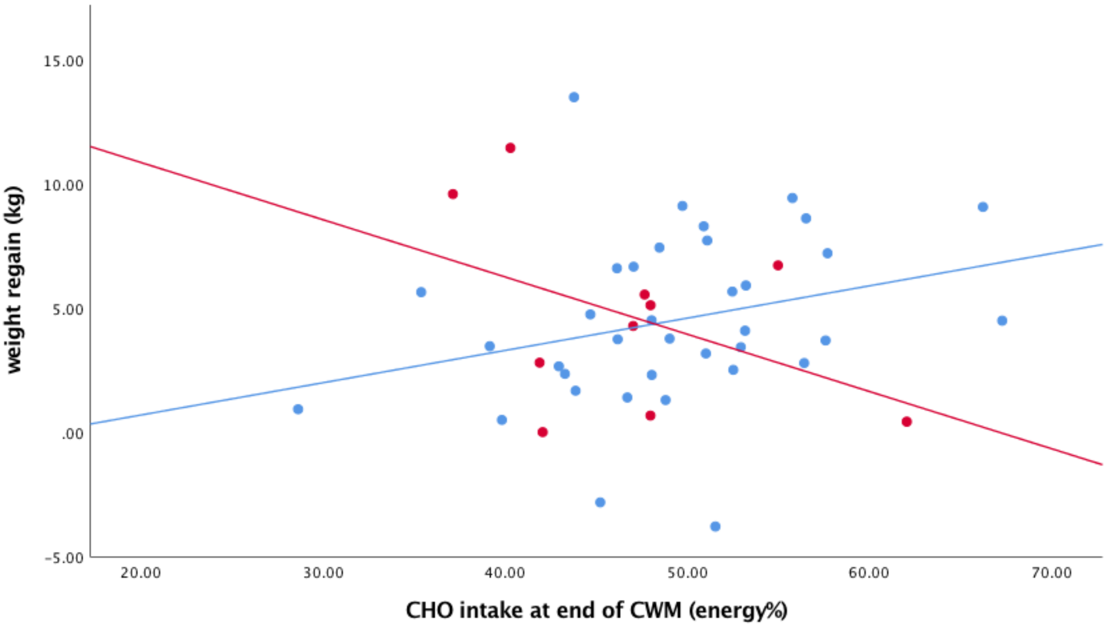

Supplemental Figure S2. Associations between carbohydrate intake at the end of CWM and weight regain in the participants with baseline normoglycemia (≤ 5.6 mmol/L) and those with baseline elevated FPG (> 5.6 mmol/L). Blue dots/line: FPG ≤ 5.6 mmol/l (regression analysis: B = 0.130, SE 0.076, p = 0.095; n = 38); red dots/line: FPG > 5.6 mmol/L (regression analysis B = -0.231, SE 0.168, p = 0.206; n = 10).

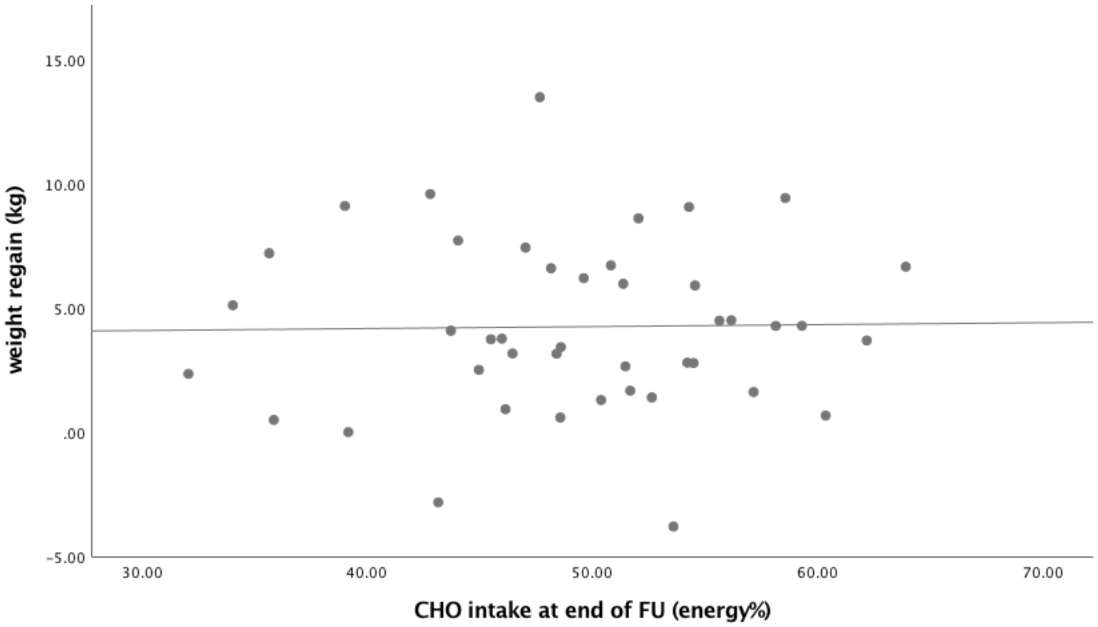

Supplemental Figure S3. Association between dietary carbohydrate (CHO) intake at the end of FU and weight regain (n = 44). Regression analysis: B = 0.008, SE 0.071, p = 0.912.

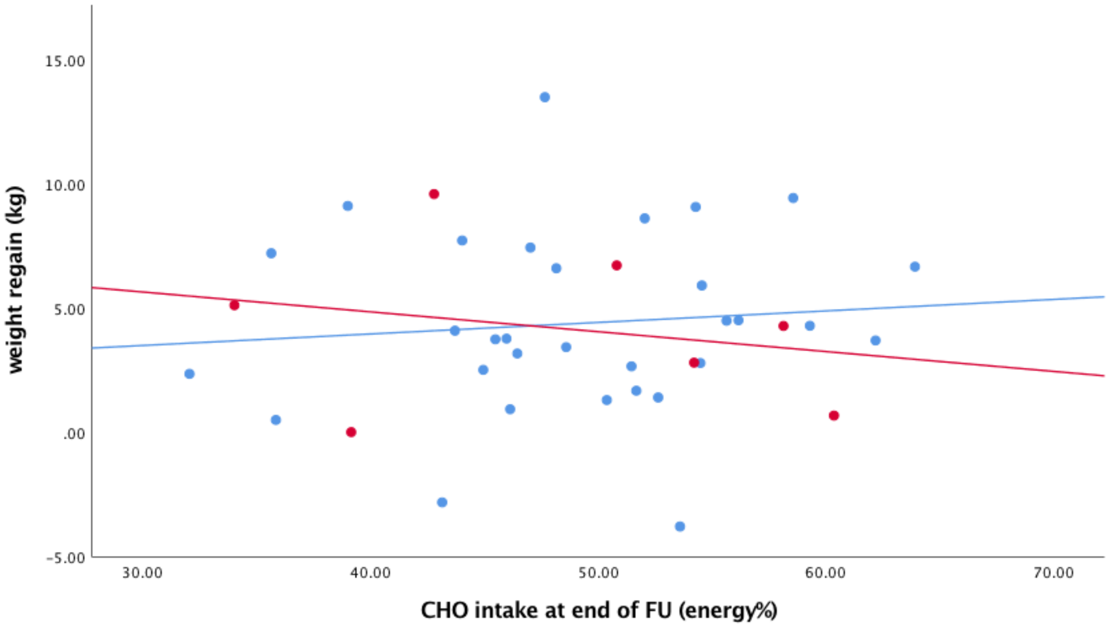

Supplemental Figure S4. Associations between carbohydrate intake at the end of FU and weight regain in the participants with baseline normoglycemia (≤ 5.6 mmol/L) and those with baseline elevated FPG (> 5.6 mmol/L). Blue dots/line: FPG ≤ 5.6 mmol/l (regression analysis: B = 0.046, SE 0.089, p = 0.607; n = 37); red dots/line: FPG > 5.6 mmol/L (regression analysis B = -0.080, SE 0.147, p = 0.609; n = 7).

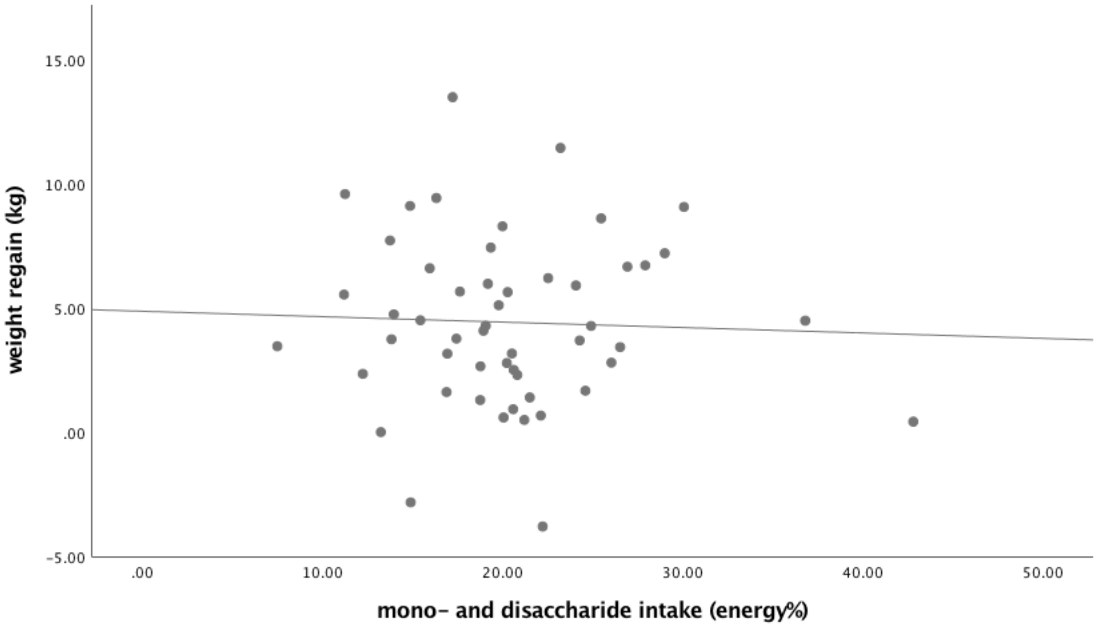

Supplemental Figure S5. Association between mono- and disaccharide intake during the FU period and weight regain (*n* = 53). Regression analysis: B = -0.022, SE 0.077, *p* = 0.777.

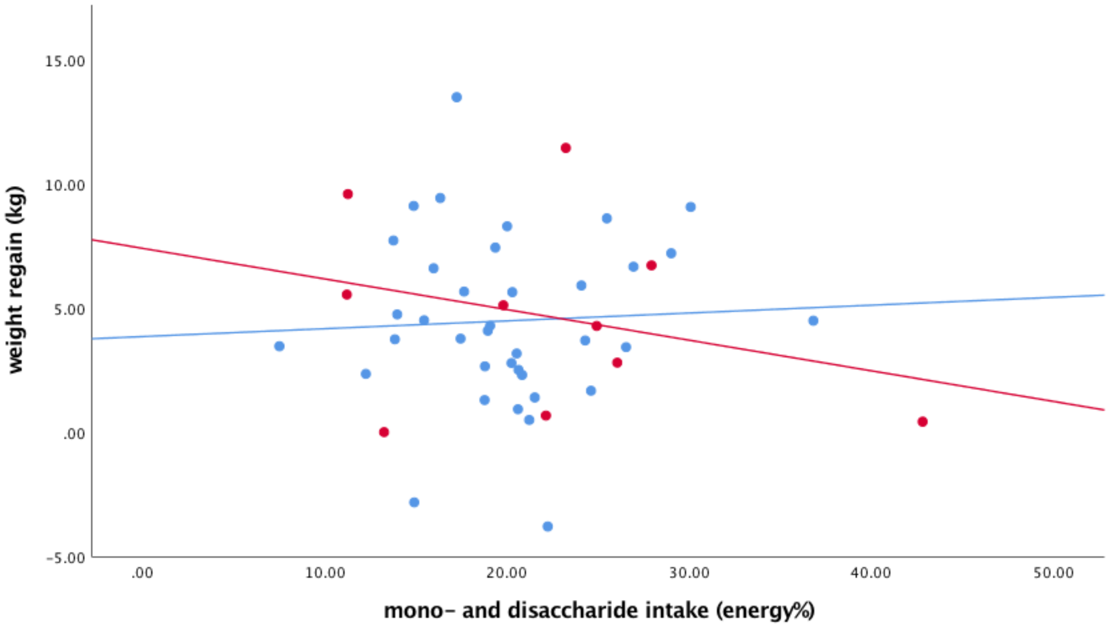

Supplemental Figure S6. Associations between mono- and disaccharide intake during the FU period and weight regain in the participants with baseline normoglycemia (≤ 5.6 mmol/L) and those with baseline elevated FPG (> 5.6 mmol/L). Blue dots/line: FPG ≤ 5.6 mmol/l (regression analysis: B = 0.032, SE = 0.104, *p* = 0.764; *n* = 39); red dots/line: FPG > 5.6 mmol/L (regression analysis B = -0.123, SE 0.138, *p* = 0.397; *n* = 10).

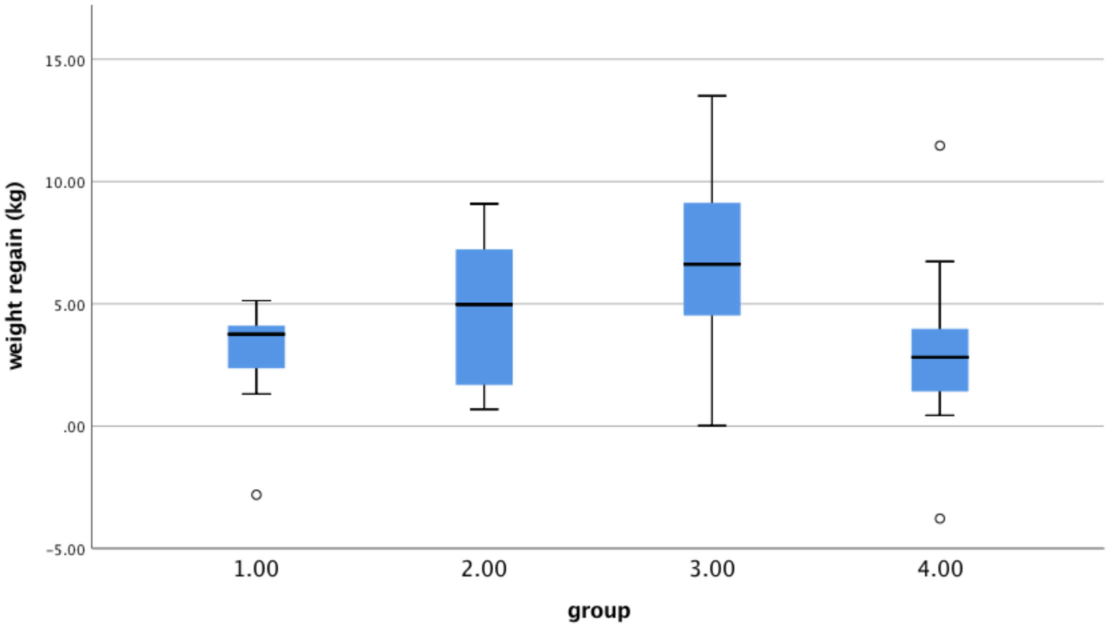

Supplemental Figure S7. Weight regain in groups of subjects with different combinations of baseline FPG and mono- and disaccharide intake. Group 1: baseline FPG < 5.6 mmol/l and mono- and disaccharide intake during the FU period ≤ median (20.0 energy%); group 2: FPG < 5.6 mmol/L and > median mono- and disaccharide intake; group 3: FPG ≥ 5.6 mmol/L and ≤ median mono- and disaccharide intake; group 4: FPG ≥ 5.6 mmol/L and > median mono- and disaccharide intake (*n* = 49).

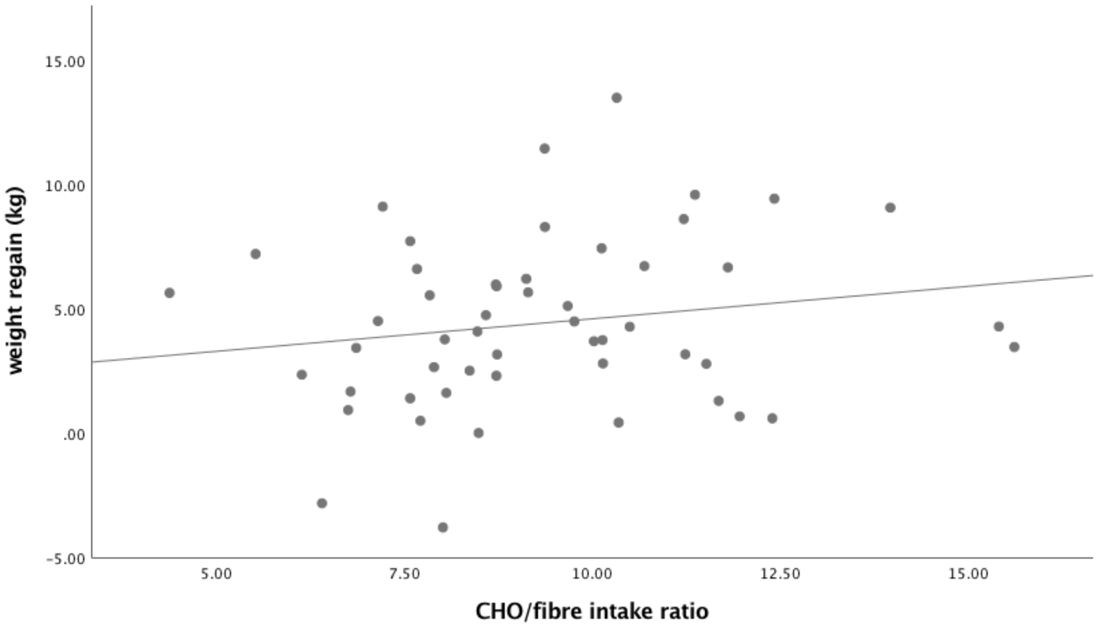

Supplemental Figure S8. Association between the CHO to fibre intake ratio during the FU period and weight regain (*n* = 53). Regression analysis: B = 0.261, SE 0.205, *p* = 0.208.

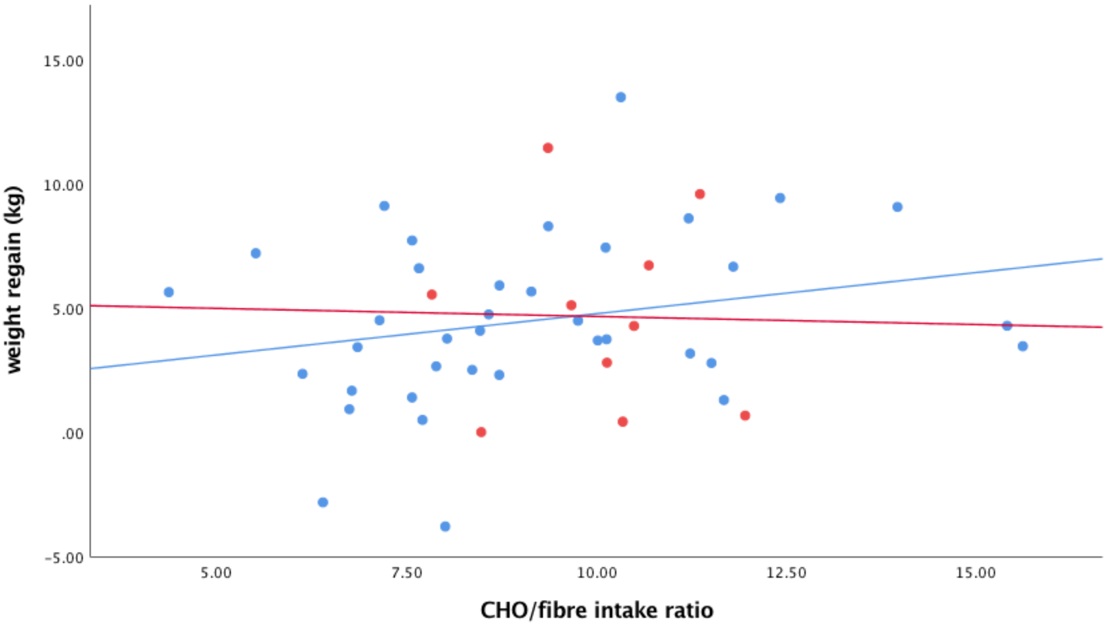

Supplemental Figure S9. Associations between CHO to fibre intake ratio during the FU period and weight regain in the participants with baseline normoglycemia (≤ 5.6 mmol/L) and those with baseline elevated FPG (> 5.6 mmol/L). Blue dots/line: FPG ≤ 5.6 mmol/l (regression analysis: B = 0.332, SE = 0.218, $p = 0.137$; $n = 39$); red dots/line: FPG > 5.6 mmol/L (regression analysis B = -0.065, SE 1.094, $p = 0.954$; $n = 10$).

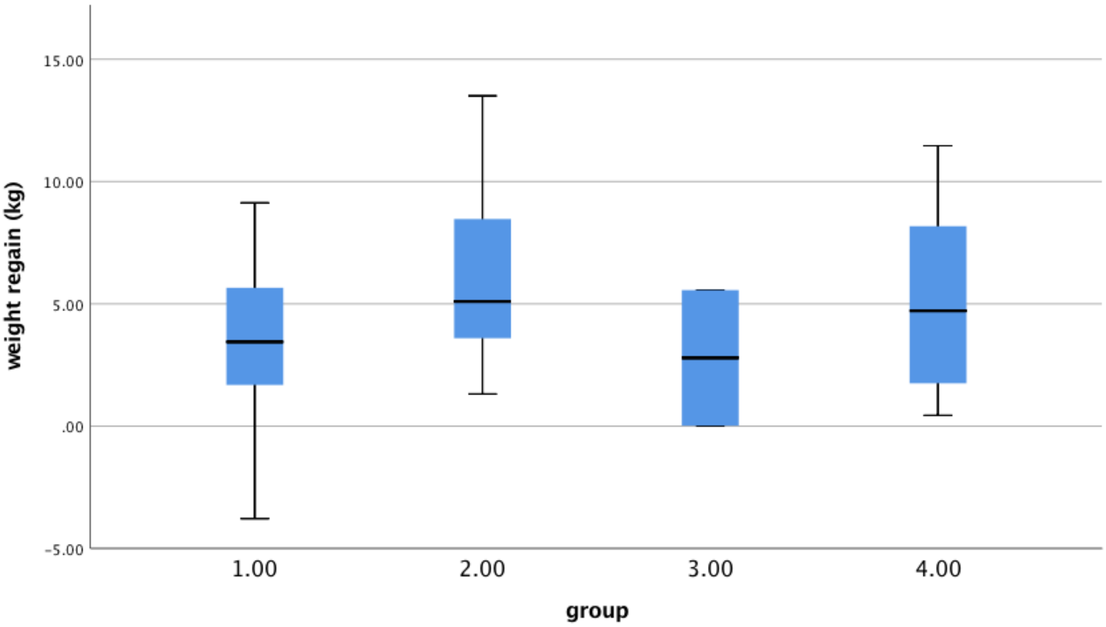

Supplemental Figure S10. Weight regain in groups of subjects with different combinations of baseline FPG and mono- and disaccharide intake. Group 1: baseline FPG < 5.6 mmol/l and CHO-to-fibre intake ratio during the FU period ≤ median (8.92); group 2: FPG < 5.6 mmol/L and > median CHO-to-fibre intake ratio; group 3: FPG ≥ 5.6 mmol/L and ≤ median CHO-to-fibre intake ratio; group 4: FPG ≥ 5.6 mmol/L and > median CHO-to-fibre intake ratio ($n = 49$).
